# Effectiveness of the use of Low Molecular Heparin in patients with repetition abortion history: Systematic review and meta-analysis

**DOI:** 10.5935/1518-0557.20200042

**Published:** 2021

**Authors:** Amanda Tamiris Barbosa Dias, Tauane Bahia Modesto, Sofia Andrade de Oliveira

**Affiliations:** 1 State University of Bahia (UNEB) - Life Sciences Department. Salvador - Bahia - Brazil

**Keywords:** Recurrent abortion, Low molecular weight heparin, Expectant conduct, Live births

## Abstract

**Objective:**

To evaluate the efficacy and safety of using low molecular weight heparin (LMWH) in women with a history of recurrent abortion without an identified cause.

**Methods:**

To develop a systematic review to select the studies. Total found 437 papers. Seven studies were completed or requested. The following variables were analyzed: clinical pregnancy, implantation rate, live births, abortion, premature birth, pregnancy, continuous pregnancy, beyond the 20^th^ gestational week, congenital abnormality, hemorrhage, preeclampsia, placental premature detachment.

**Results:**

The LMWH group had a higher incidence of continuous pregnancy after the 20^th^ week of gestation and there was no significant difference between the LMWH group and the expectant management group in the analysis of other variables.

**Conclusions:**

There was no data showing risk and/or less use of LMWH compared to expectant management; on the contrary, LMWH use increased the incidence of evolutionary pregnancy after the 20^th^ gestational week. LMWH has some influence on prophylactic treatment of repeat abortion for unknown cause.

## INTRODUCTION

Repeat abortion, also called habitual gestational loss, was defined, according to ^Stirrat (1990)^, as the successive spontaneous cessation of three or more pregnancies with 20-22 gestational weeks or less ^(Montenegro & Rezende Filho, 2008)^. However, according to the Practice Committee of the American Society for Reproductive Medicine (2008), habitual abortion is considered when there is two or more consecutive gestational losses.

Habitual gestational loss is a common obstetric complication that affects about 5% of pregnant women ^(Practice Medicine, 2012^; ^Rai & Regan, 2006)^. There are Several factors that may be involved in the etiology of recurrent miscarriages, including genetic, anatomical (arcuate, bicornuate, septate, T-shaped and unicornuate uterus), endocrine abnormalities (luteal phase insufficiency), hyperprolactinemia, thyroid disease, polycystic ovary syndrome, immunological, infectious, metabolic, thrombophilia and maternal age ^(Burlá et al., 2015)^. However, around 50% of cases have no identified cause ^(Practice Committee of the American Society for Reproductive Medicine, 2012)^.

Considering that episodes of thrombosis in the uteroplacental circulation occur independently of the presence of thrombophilia ^(Badawy et al., 2008^; ^Nelson & Greer, 2008)^, some authors began to institute empirical prophylactic treatment with low molecular weight heparin (LMWH) and/or small doses of aspirin in women with habitual miscarriage of unexplained cause ^(Badawy *et al*., 2008^; ^Pasquier et al., 2015)^. Although there are already studies indicating the benefit of thromboprophylaxis ^(Badawy *et al*., 2008^; ^Fawzy et al., 2008^; ^Shaaban et al., 2017^; ^Urman et al., 2009)^, there are also studies showing that there are no significant differences in the outcome of pregnancies of women undergoing LMWH. Therefore, this strategy should not be instituted as routine practice until there are studies that prove the exact mechanism of action and the benefit of this therapy in repetitive abortions ^(Pasquier *et al*., 2015^; ^Schleussner et al., 2015^; ^Di Nisio et al., 2005^; ^Kaandorp et al., 2010^; ^Clark et al., 2010^; ^de Jong et al., 2014^; ^Visser etal., 2011)^.

Failure to implant in couples undergoing assisted reproduction is also a relatively common phenomenon that affects their feelings of frustration and despair. Implantation failure, as well as habitual abortion, has been attributed to a number of factors; however, most have no particular cause. Knowledge about the contribution of coagulation disorders in the implantation failure process underlies the use of anticoagulants during assisted reproduction therapy ^(Urman et al., 2009)^. The success of this therapy can be assessed by the implantation rate, which is defined as the number of observed gestational sacs divided by the number of embryos transferred ^(Zegers-Hochschild et al., 2009)^.

Thus, considering the existence of still inconclusive studies and, knowing the emotional repercussions of spontaneous abortion that involves feelings of loss and blame for the impossibility of completing the pregnancy, this requires adequate, safe and humanized technical attention. This study aims to evaluate the efficacy and safety of LMWH use in women with a history of recurrent miscarriage without an identified cause.

## MATERIAL AND METHODS

### Evidence Acquisition

To describe the results of this meta-analysis, we used the Preferred Reporting Items for Systematic Reviews and Meta-Analyzes (PRISMA) ^(Moher et al., 2009)^. This systematic review is registered in the PROSPERO database under registration number: CRD42017082373.

### Study goal

To establish the focus of the systematic review, we used the following clinical issues: the population studied, the intervention and comparisons, study design and study results from which we extracted the data. Thus, the goal of this systematic review was “to detect the efficacy of LMWH in the prophylactic treatment of women with recurrent abortion compared with the expectant management”. We used only randomized and quasi-randomized studies, and we structured the meta-analysis following the “PICOS” format (Population, Intervention, Comparison, Results, Type of study) ^(Howick, 2009)^ (Table 1).

**Table 1:** Selection criteria for the studies included (PICOS)

	Included	Excluded
**Population**	Women with repeat abortion.	
**Intervention**	Low molecular weight heparin treatment	
**Comparison**	Expectant Conduct	
**Results**	Primary: Live births, spontaneous abortion, continuous pregnancy beyond the 20^th^ gestational week, implantation rate.	
	Secondary: clinical pregnancy, premature birth, multiple pregnancy, congenital abnormality, hemorrhage, preeclampsia, placental premature detachment.	
**Type of study**	Double-blind and quasi randomized studies	Systematic review and meta-analyzes, case control study, case reports, comments, nonrandomized

- **P:** Women with repeat abortion history.
- **I:** Low molecular weight heparin treatment
- **C:** Expectant conduct
- **O:** Effectiveness
- **S:** Randomized and quasi-randomized studies

### Eligibility criteria

The selected studies had to include women with recurrent abortion with two or more consecutive gestational losses; to compare the efficacy of LMWH *versus* the expectant management in prophylactic treatment of habitual abortion, and to be a randomized or quasi-randomized trial.

The studies were not included if they were published in summary format, letter to the editor, comments, meta-analysis, review article, or studies that included any drug other than LMWH in the study population that included women with repeat abortion of known cause.

### Study strategy (Appendix 1)

We carried out an electronic search in MEDLINE and PubMed in October 2017. There was no language restriction for the papers. We based the study on the following combined Medical Subject Headings of the National Library of Medicine (MeSH) terms: "habitual abortion", "anticoagulants", "watchful waiting", "gestational age", "fetal death", "live births", "premature birth", "prolonged pregnancies", "perinatal death", "intrauterine growth retardation", "congenital abnormality", "obstetric complications", "pregnancy associated hypertension", "pre-eclampsia", "placenta previa", "placental abruption", "uterine hemorrhage", "postpartum hemorrhage".

### Study selection

Two researchers (ATBD and SAO) selected the publications independently. Initially, we assessed the titles and abstracts of all the studies found by the research strategy. Any divergence in study selection and/or data extraction was cleared by consensus between the two researchers. We read the papers that had insufficient information in the title and abstract in their entirety. Only studies that had the inclusion criteria and did not meet the non-inclusion criteria were selected for the meta-analysis. We generated a list of potential studies for inclusion in the systematic review. We checked references from reviews and meta-analyzes to find papers that could possibly meet the inclusion criteria.

### Data collection process

Two researchers (ATBD and SAO) independently extracted data using a standardized form and, again, disagreements were solved by consensus. We extracted and combined the data from all included items reporting intervention and patient outcomes. These authors evaluated the eligibility and quality of the studies and, subsequently, extracted data from the papers. The standardized form included information such as authors, journal, year of publication, design, duration and place of study, demographics of participants, inclusion and exclusion criteria, type of interventions, and outcomes.

### Data and results

We combined the studies in groups, according to the interventions performed and the outcomes found. We combined the data, to run the following analyzes:

- Is LMWH efficient for prophylactic treatment of recurrent abortion?
- What is the best conduct for prophylactic treatment of habitual abortion?
- Which approach causes fewer adverse effects as congenital abnormality, bleeding, preeclampsia and placental premature detachment?
- Which conduct best prolongs pregnancy beyond 20 gestational weeks?
- What is the best conduct to increase implantation rate?
- Which conduct generates the most premature births?
- Which conduct contributes to the higher number of multiple pregnancies?

### Partiality assessment risk

We followed the guidelines suggested by the Cochrane Collaboration group to assess the risk of partiality studies ^(Higgins & Green, 2011)^. Sequence generation, allocation concealment, blinding, and incomplete outcome data were evaluated for each trial included in the review. A low risk of bias was considered when a "yes" judgment for all domains was obtained, while a high risk of bias was considered when a "no" judgment for one or more domains was obtained. Table 2 depicts the quality assessment of the included trials.

**Table 2:** Quality assessment of included trials

Study	Random sequence generation	Allocation Concealment	Blinding	Incomplete outcomes
Badawy	Yes	Yes	No	Uncertain
Pasquier	Yes	Yes	Yes	Yes
Shaaban	Yes	Yes	Uncertain	Yes
Urman	Yes	Yes	Uncertain	Yes
Schleussner	Yes	Yes	Uncertain	Yes
Noci	Yes	Yes	Uncertain	Uncertain
Berker	Quasi-randomized	Uncertain	No	Uncertain

### Analysis

To accomplish the meta-analysis, we used the Cochrane Collaboration's Review Manager Software (RevMan 5.3; <http://tech.cochrane.org/revman>). The metanalytic measure of interest is the odds ratio, which we obtained using the Mantel-Haenszel method. In cases where the number of events in one of the groups was zero, we used the Peto's method. In addition to the odds ratios, the respective 95% confidence intervals (CI) as well as the forest plot were presented. We assessed the heterogeneity between the studies by the Higgins and Thompson I^2^ statistics and the Cochran Q test. We applied the random effect model when the I^2^ statistic was higher than 50%, or when the null hypothesis of the Cochran Q test was rejected. The statistical tests applied were bilateral and the adopted significance level was 5%.

**Figure 1:**
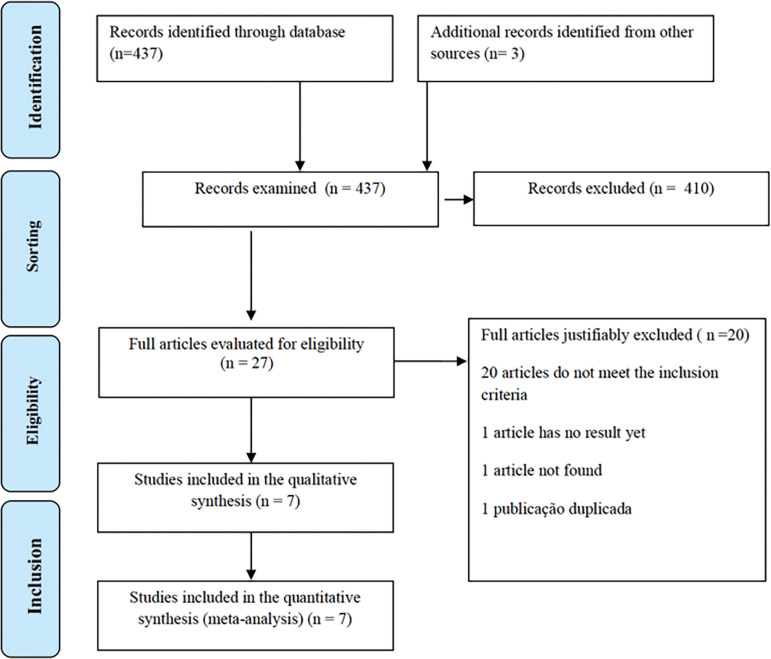
Study Selection Flowchart

We conducted a systematic literature search to identify randomized and quasi-randomized trials comparing LMWH use and expectant conduct in the prophylactic treatment of repeat abortion. In total, we found 437 papers as we can see in Figure 1. At the end of the review process, 7 papers met the inclusion criteria and were described and evaluated ^(Badawy et al., 2008^; ^Pasquier et al., 2015^; ^Shaaban et al., 2017^; ^Urman et al., 2009^; ^Schleussner et al., 2015^; ^Berker et al., 2011^; ^Noci et al., 2011)^ (Fig. 1). There were six randomized studies and one quasi-randomized study. We excluded 433 studies, because they either did not meet the inclusion criteria or did not provide sufficient data for inclusion in the meta-analysis, or were literature review studies. From these studies, 280 evaluated women with a determined cause of miscarriage as thrombophilia, 80 included aspirin with or without LMWH in the intervention group or had women on aspirin as a control group. We added three studies identified in the meta-analyses’ references that met the inclusion criteria. We also included studies evaluating the efficacy of LMWH in the prophylactic treatment of repeat abortion in pregnant women by in vitro fertilization. Table 2 shows the quality assessment of the included studies.

## RESULTS

## Description of included studies (Appendix 2)

The included studies represented 1,855 patients (936 undergoing LMWH and 919 in the expectant management group). Appendix 1 shows the summary characteristics of the studies included in this review. Four studies ^(Badawy et al., 2008^; ^Pasquier et al., 2015^; ^Shaaban et al., 2017^; ^Schleussner et al., 2015)^ evaluated the use of LMWH prevention of indeterminate recurrent miscarriage and the other three studies ^(Urman et al., 2009^; ^Berker et al., 2011^; ^Noci et al., 2011)^ evaluated the effect of LMWH on implantation rates in women with recurrent implantation failure (RIF), but without coagulation disorders.

### Clinical pregnancy

**Figure 2:**
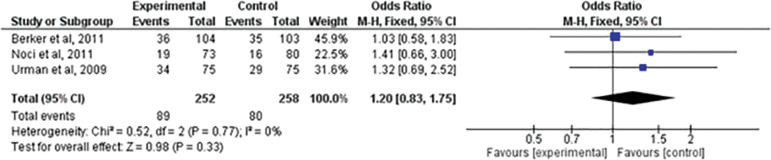
Forest-plot of clinical pregnancy incidence with treatment

We assessed the number of women who reached clinical pregnancy after treatment. We analyzed this in only three articles ^(Berker et al., 2011^; ^Noci et al., 2011^; ^Urman et al., 2009)^. When comparing LMWH *versus* expectant management, there was no statistically significant difference between the groups (RR=1.20; 95% CI: 0.83, 1.75; I^2^=0%; *p=*0.33) (Fig. 2).

### Implantation rate

**Figure 3:**
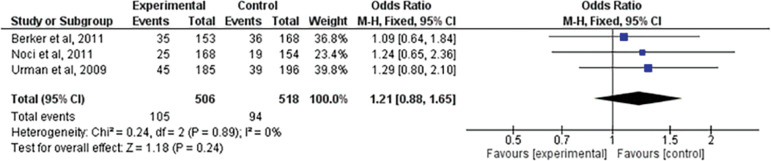
Forest-plot of implantation rate incidence with treatment

Three studies ^(Berker et al., 2011^; ^Noci et al., 2011^; ^Urman et al., 2009)^ evaluated the implantation rate after treatment. When comparing the two interventions there was no statistically significant difference (RR=1.21; 95% CI: 0.88, 1.65; I^2^=0%; *p=*0.24) (Fig. 3).

### Live births

**Figure 4:**
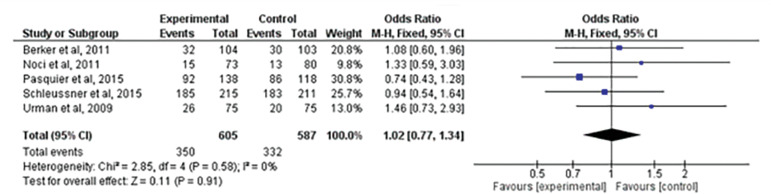
Forest-plot of live birth incidence with treatment

There were five studies ^(Berker et al., 2011^; ^Noci et al., 2011^; ^Pasquier et al., 2015^; ^Schleussner et al., 2015^; ^Urman et al., 2009)^ that evaluated the incidence of live births after treatment. When comparing the five studies, we found no significant static difference between the groups (RR=1.02; 95% CI: 0.77, 1.34; I^2^=0%; *p=*0.91) (Fig. 4).

### Spontaneous abortion

**Figure 5:**
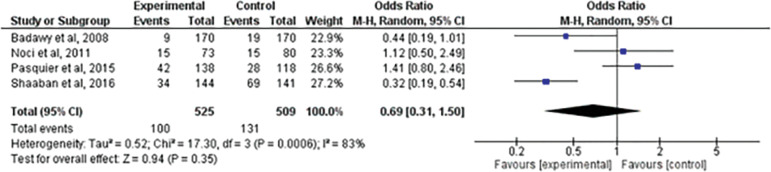
Forest-plot of spontaneous abortion incidence with treatment

We assessed the presence of spontaneous abortion with the institution of treatment. Four papers evaluated this outcome ^(Badawy et al., 2008^; ^Noci et al., 2011^; ^Pasquier et al., 2015^; ^Shaaban et al., 2017)^. When comparing the two interventions, there was no statistically significant difference between the groups (RR=0.69; 95% CI: 0.31, 1.50; I^2^=83%; *p=*0.35) (Fig. 5).

### Premature birth

**Figure 6:**
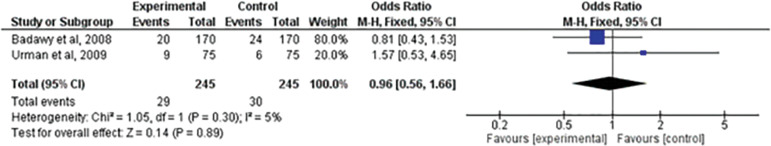
Forest-plot of premature birth incidence with treatment

We evaluated the number of premature births that occurred after treatment. It was possible to analyze it in only two studies ^(Badawy et al., 2008^; ^Urman et al., 2009)^. When comparing LMWH *versus* expectant management, there was no statistically significant difference between the groups (RR=0.96; 95% CI: 0.56, 1.66; I^2^=5%; *p=*0.89) (Fig. 6).

### Multiple pregnancy

**Figure 7:**
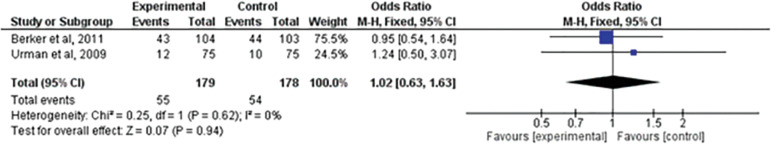
Forest-plot of multiple pregnancies incidence with treatment

We could analyze only two papers ^(Berker et al., 2011^; ^Urman et al., 2009)^. When comparing the two treatments, there was no statistically significant difference between the groups (RR=1.02; 95% CI: 0.63, 1.63; I^2^=0%; *p=*0.94) (Fig. 7).

### Continuous pregnancy beyond the 20th gestational week

**Figure 8:**
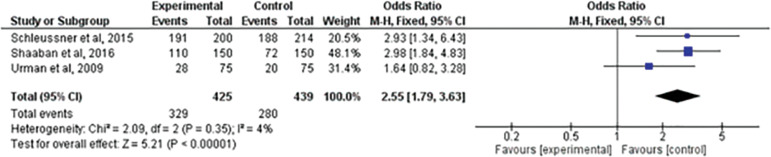
Forest-plot of continuous pregnancy beyond the 20^th^ gestational week incidence with treatment

Three studies ^(Schleussner et al., 2015^; ^Shaaban et al., 2017^; ^Urman et al., 2009)^ evaluated the incidence of continuous pregnancy beyond the 20^th^ gestational week with the institution of treatment. When comparing the two treatments, there was a statistically significant difference between the groups (RR=2.55; 95% CI: 1.79, 3.63; I^2^=4%; *p=*0.00001). Treatment with low molecular weight heparin resulted in a higher incidence of continuous pregnancy beyond the 20^th^ gestational week (Fig. 8).

### Congenital abnormality

**Figure 9:**
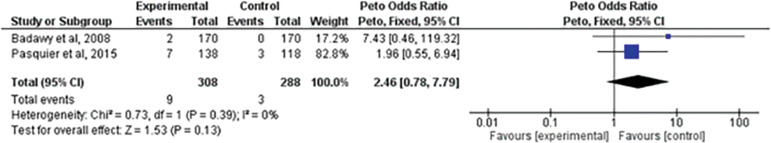
Forest-plot of congenital abnormality incidence with treatment

Two studies ^(Badawy et al., 2008^; ^Pasquier et al., 2015)^ evaluated the incidence of congenital abnormality after treatment. When comparing the two interventions, there was no statistically significant difference (RR=2.46; 95% CI: 0.78, 7.79; I^2^=0%; *p=*0.13) (Fig. 9).

### Hemorrhage

**Figure 10:**
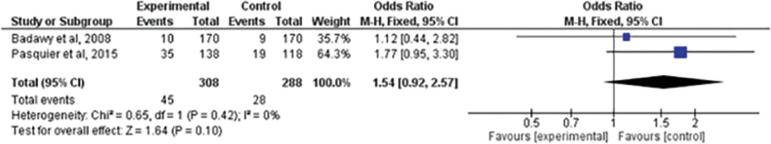
Forest-plot of hemorrhage incidence with treatment

Two studies ^(Badawy et al., 2008^; ^Pasquier et al., 2015)^ evaluated the incidence of hemorrhage after treatment. When comparing the two studies, there was no statistically significant difference between the groups (RR=1.54; 95% CI: 0.92, 2.57; I^2^=0%; *p=*0.10) (Fig. 10).

### Preeclampsia

**Figure 11:**
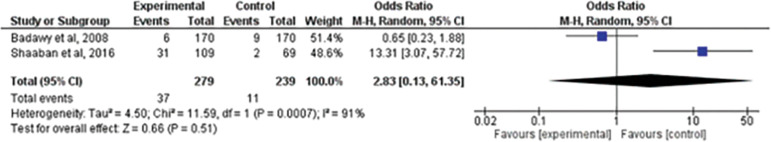
Forest-plot of preeclampsia incidence with treatment

We could analyze only two studies regarding the incidence of preeclampsia ^(Badawy et al., 2008^; ^Shaaban et al., 2017)^. When comparing LMWH *versus* expectant management there was no statistically significant difference between the groups (RR=2.83; 95% CI: 0.13, 61.35; I^2^=91%; *p=*0.51) (Fig. 11).

### Placental premature detachment

**Figure 12:**
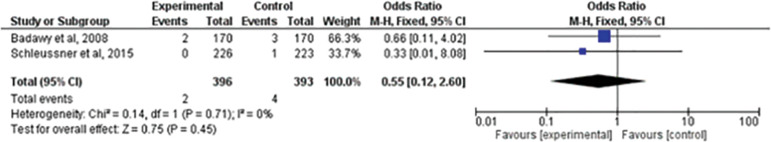
Forest-plot of placental premature detachment incidence with treatment

We evaluated the presence of placental premature detachment as a side effect during treatment. Only two papers evaluated this outcome ^(Badawy et al., 2008^; ^Schleussner et al., 2015)^. When comparing the two interventions, there was no statistically significant difference between the groups (RR=0.55; 95% CI: 0.12, 2.60; I^2^=0%; *p=*0.45) (Fig. 12).

## DISCUSSION

In this study, we demonstrated evidence of LMWH not being inferior to the expectant management. The LMWH group had a higher incidence of evolutionary pregnancy beyond the 20^th^ gestational week without including unfavorable factors during pregnancy, since there was no increase in variants such as hemorrhage, preeclampsia, placental premature detachment and preeclampsia in this group. However, it is important to highlight that this outcome was evaluated in only three studies ^(Shaaban et al., 2017^; ^Urman et al., 2009^; ^Schleussner et al., 2015)^, which demonstrates the need for a larger number of studies evaluating pregnancies that continue beyond the 20^th^ gestational week.

The rate of spontaneous abortion is a variable of great interest when assessing prophylactic treatments for recurrent miscarriage, and in this study this variable showed no statistically significant difference (RR=0.69; 95% CI: 0.31, 1.50 (83%; *p=*0.35). However, there is a tendency towards favoring LMWH, but the four studies analyzed ^(Badawy et al., 2008^; ^Pasquier et al., 2015^; ^Shaaban et al., 2017^; ^Noci et al., 2011)^ have a high heterogeneity rate (I^2^=83%), thus requiring more studies for the conclusion to become more reliable.

The use of LMWH showed no statistically significant difference in the implantation rate analysis, when compared to the expectant management (RR=1.21; 95% CI: 0.88, 1.65; I^2^=0%; *p=*0.24). However, only three studies ^(Urman et al., 2009^; ^Berker et al., 2011^; ^Noci et al., 2011)^ were evaluated by the meta-analysis, indicating that there is also a need for further studies to reach a more reliable conclusion about the action and safety of using LMWH to treat implantation failures.

The preeclampsia variable showed a high heterogeneity between studies (I^2^=91%). For this variable, two studies were evaluated ^(Badawy et al., 2008^; ^Shaaban et al., 2017)^. In the first, the control group had a higher incidence of preeclampsia compared to the intervention group, unlike the second study in which the highest incidence of preeclampsia occurred in the intervention group. The presence of high heterogeneity of this variable makes it questionable the combination of the results of these studies for this outcome.

In their meta-analysis, ^Di Nisio et al. (2005)^ compared aspirin, unfractionated heparin and LMWH use compared with one another, or a placebo to prevent birth loss in pregnant women or women who were trying to become pregnant and who had a history of at least two consecutive abortions with no apparent causes. One of the included studies ^(Gris et al., 2004)^ resulted in increased live birth rates compared to low aspirin doses. Four patients had preeclampsia in the enoxaparin intervention group, and three participants in the aspirin group had it. There was one case of premature birth in the aspirin intervention group. However, since the studies included in the meta-analysis had a small sample size and methodological limitations, they concluded that thromboprophylaxis should not be prescribed until convincing data exists.

There was a meta-analysis in 2009 ^(Kaandorp et al., 2009)^, evaluating the efficacy and safety of aspirin, LMWH and fractional heparin compared with one another or with placebo in women with a history of at least two miscarriages of spontaneous causes. The rate of live births was similar between the enoxaparin group (82%) and the aspirin group (84%) (RR 0.97; 95% CI: 0.81 to 1.16). Three women had preeclampsia in the aspirin group and no women had preeclampsia in the enoxaparin group. In each group, there was one birth with congenital abnormalities. None of the studies showed greater efficacy of one treatment over the other, so the meta-analysis concluded that the use of anticoagulants should not be recommended.

In 2014, nine studies included in a meta-analysis ^(de Jong et al., 2014)^ reviewed the effects of LMWH, fractional heparin or aspirin, or a combination of both compared with one another or to placebo in the prophylactic treatment of pregnant women with a history of at least two miscarriages. There were three studies assessing the LMWH effects ^(Badawy et al., 2008^; ^Fawzy et al., 2008^; ^Martinelli et al., 2012)^. There were no differences between treatment groups among individual studies for gestational complications, bleeding or thromboembolic events. Based on these results, the review showed that the use of anticoagulants in women with recurrent miscarriage was not effective.

Although these meta-analyses do not specifically compare LMWH and expectant management, they are important because they show in their results that patients who received prophylactic LMWH treatment had statistically insignificant results when compared to other treatments, which differs from our study, that already presents statistically significant differences favoring the LMWH group when analyzing the continuous pregnancy beyond the 20^th^ gestational week variable.

We believe this study contributes to the still unresolved debate about the use of LMWH in the prophylactic treatment of recurrent miscarriage. When comparing this intervention with expectant management, there was no data to show risk and/or lower efficacy of LMWH. On the contrary, LMWH was more effective in increasing the incidence of evolutionary pregnancy beyond the 20^th^ gestational week; thus indicating that LMWH has some influence on the prophylactic treatment of recurrent miscarriage of unknown cause.

Therefore, we need further studies with standardized methods to evaluate the comparison of LMWH and expectant management.

## Appendix 1. Research Strategy

2 Abortions, Habitual) OR (Habitual Abortion) OR (Habitual Abortions) OR (Miscarriage, Recurrent) OR (Miscarriages, Recurrent) OR (Recurrent Miscarriage) OR (Recurrent Miscarriages) OR (Abortion, Recurrent) OR (Abortions, Recurrent) OR (Recurrent Abortion) OR (Recurrent Abortions)

5 (Anticoagulation Agents) OR (Agents, Anticoagulation) OR (Anticoagulant Agents) OR (Agents, Anticoagulant) OR (Anticoagulant Drugs) OR (Drugs, Anticoagulant) OR (Anticoagulant) OR (Indirect Thrombin Inhibitors) OR (Inhibitors, Indirect Thrombin) OR (Thrombin Inhibitors, Indirect)

7(Waiting, Watchful) OR (Watchful Waiting) OR (Watchful Waiting)

10 (Age, Gestational) OR (Ages, Gestational) OR (Gestational Ages) OR (Maturity, Chronologic Fetal) OR (Chronologic Fetal Maturity) OR (Fetal Maturity, Chronologic) OR (Fetal Age) OR (Age, Fetal) OR (Ages, Fetal) OR (Fetal Ages)

13 (Fetal Death) OR (Deaths, Fetal) OR (Fetal Deaths) OR (Fetal Demise) OR (Demise, Fetal) OR (Fetal Mummification) OR (Mummification, Fetal)

15 (Live Births)

17 (Birth, Premature) OR (Births, Premature) OR (Premature Births) OR (Preterm Birth) OR (Birth, Preterm) OR (Births, Preterm) OR (Preterm Births)

19 (Pregnancies, Prolonged) OR (Prolonged Pregnancies) OR (Prolonged Pregnancy)

21 (Death, Perinatal) OR (Deaths, Perinatal) OR (Perinatal Deaths) OR (Neonatal Death) OR (Death, Neonatal) OR (Deaths, Neonatal) OR (Neonatal Deaths)

24 (Growth Retardation, Fetal) OR (Retardation, Fetal Growth) OR (Intrauterine Growth Retardation) OR (IUGR) OR (Growth Retardation, Intrauterine) OR (Retardation, Intrauterine Growth)

27 (Abnormality, Congenital) OR (Congenital Abnormality) OR (Deformities) OR (Deformity) OR (Congenital Defects) OR (Congenital Defect) OR (Defect, Congenital) OR (Defects, Congenital) OR (Abnormalities, Congenital) OR (Birth Defects) OR (Birth Defect) OR (Defect, Birth) OR (Defects, Birth)

29 (Complication, Obstetric Labor) OR (Complications, Obstetric Labor) OR (Labor Complication, Obstetric) OR (Labor Complications, Obstetric) OR (Obstetric Labor Complication) OR (Labor Complications) OR (Complication, Labor) OR (Labor Complication) OR (Complications, Labor)

31 (Hypertension, Pregnancy Induced) OR (Pregnancy-Induced Hypertension) OR (Pregnancy Induced Hypertension) OR (Hypertensions, Pregnancy Induced) OR (Induced Hypertension, Pregnancy) OR (Induced Hypertensions, Pregnancy) OR (Gestational Hypertension) OR (Hypertension, Gestational) OR (Transient Hypertension, Pregnancy) OR (Hypertension, Pregnancy Transient) OR (Pregnancy Transient Hypertension)

33 (Pre-Eclampsia) OR (Preeclampsia) OR (Pregnancy Toxemias) OR (Pregnancy Toxemia) OR (Toxemia, Pregnancy) OR (Edema-Proteinuria-Hypertension Gestosis) OR (Edema Proteinuria Hypertension Gestosis) OR (Gestosis, Edema-Proteinuria-Hypertension) OR (Hypertension-Edema-Proteinuria Gestosis) OR (Gestosis, Hypertension-Edema-Proteinuria) OR (Hypertension Edema Proteinuria Gestosis) OR (Toxemia Of Pregnancy) OR (Of Pregnancies, Toxemia) OR (Of Pregnancy, Toxemia) OR (Pregnancies, Toxemia Of) OR (Pregnancy, Toxemia Of) OR (Toxemia Of Pregnancies) OR (EPH Complex) OR (EPH Toxemias) OR (EPH Toxemia) OR (Toxemia, EPH) OR (Toxemias, EPH) OR (EPH Gestosis) OR (Gestosis, EPH) OR (Toxemias, Pregnancy) OR (Preeclampsia Eclampsia 1) OR (1, Preeclampsia Eclampsia) OR (1s, Preeclampsia Eclampsia) OR (Eclampsia 1, Preeclampsia) OR (Eclampsia 1s, Preeclampsia) OR (Preeclampsia Eclampsia 1s) OR (Proteinuria-Edema-Hypertension Gestosis) OR (Gestosis, Proteinuria-Edema-Hypertension) OR (Proteinuria Edema Hypertension Gestosis)

36 (Placental Abruption) OR (Abruption, Placental) OR (Abruptions, Placental) OR (Placental Abruptions)

38 (Uterine Hemorrhages) OR (Hemorrhage, Uterine) OR (Uterine Bleeding) OR (Bleeding, Uterine) OR (Uterine Bleedings) OR (Vaginal Bleeding) OR (Bleeding, Vaginal) OR (Bleedings, Vaginal) OR (Vaginal Bleedings)

40 (Hemorrhage, Postpartum) OR (Immediate Postpartum Hemorrhage) OR (Hemorrhage, Immediate Postpartum) OR (Postpartum Hemorrhage, Immediate) OR (Delayed Postpartum Hemorrhage) OR (Hemorrhage, Delayed Postpartum) OR (Postpartum Hemorrhage, Delayed)

41(2 AND 5) AND 10

42(2 AND 5) AND 13

43(2 AND 5) AND 15

44(2 AND 5) AND 17

45(2 AND 5) AND 19

46(2 AND 5) AND 21

47(2 AND 5) AND 24

48(2 AND 5) AND 27

49(2 AND 5) AND 29

50(2 AND 5) AND 31

51(2 AND 5) AND 33

52(2 AND 5) AND 36

53(2 AND 5) AND 38

54(2 AND 5) AND 40

55(2 AND 7) AND 10

56(2 AND 7) AND 13

58(2 AND 7) AND 15

59(2 AND 7) AND 15 Schema: all

60(2 AND 7) AND 17

61(2 AND 7) AND 19

62(2 AND 7) AND 21

63(2 AND 7) AND 21 Schema: all

64(2 AND 7) AND 24

65(2 AND 7) AND 24 Schema: all

66(2 AND 7) AND 27

67(2 AND 7) AND 27 Schema: all

68(2 AND 7) AND 29

69(2 AND 7) AND 31

70(2 AND 7) AND 31 Schema: all

71(2 AND 7) AND 33

72(2 AND 7) AND 33 Schema: all

73(2 AND 7) AND 36

74(2 AND 7) AND 36 Schema: all

75(2 AND 7) AND 38

76(2 AND 7) AND 38 Schema: all

77(2 AND 7) AND 40

78(2 AND 7) AND 40 Schema: all

79((2 AND 5) AND 10)) OR ((2) AND 5) AND 13)) OR ((2) AND 5) AND 15)) OR ((2) AND 5) AND 17)) OR ((2) AND 5) AND 19)) OR ((2) AND 5) AND 21)) OR ((2) AND 5) AND 24)) OR ((2) AND 5) AND 27)) OR ((2) AND 5) AND 29)) OR ((2) AND 5) AND 31)) OR ((2) AND 5) AND 33)) OR ((2) AND 5) AND 36)) OR ((2) AND 5) AND 38)) OR ((2) AND 5) AND 40)) OR ((2) AND 7) AND 10)) OR ((2) AND 7) AND 15)) OR ((2) AND 7) AND 17)) OR ((2) AND 7) AND 19)) OR ((2) AND 7) AND 21)) OR ((2) AND 7) AND 24)) OR ((2) AND 7) AND 27)) OR ((2) AND 7) AND 29)) OR ((2) AND 7) AND 31)) OR ((2) AND 7) AND 33)) OR ((2) AND 7) AND 36)) OR ((2) AND 7) AND 38)) OR ((2) AND 7) AND 40)) OR ((2) AND 7) AND 13)

## Appendix 2. Characteristics of included studies

**Badawy *et al*., 2008**

**Table:**

Authors	A.M. Badawy, M. Khiary, L. S. Sherif, M. Hassan, A. Ragab, I. Abdelall
Journal	Journal of de Obstetrics and Gynecology
Year of publication	2008
Country	Egypt
Period	From February, 2003 to January, 2006
Study Designer	Prospective randomized study
Randomization	Women were randomly allocated to either group A (thromboprophylaxis group, n=170) or group B (control group n=170) using pre-filled sealed envelopes designed by the investigators for each patient.
Inclusion criteria	Pregnant women before 8 weeks of gestation with a history of three or more consecutive first trimester abortion (first 12 weeks of gestation) with no identifiable etiology after full investigations. Habitual abortion was defined as a history of three or more consecutive abortions in the first trimester.
Exclusion criteria	Women with any identifiable etiology for spontaneous abortions, such as hereditary thrombophilia.
Participant demographic data	There are no significant differences between the two groups. The age of the patients in the study ranged from 18 to 36 years. A total of 52 patients (30.5%) in group A and 45 patients (26.4%) in group B had deliveries before completing the full term. There was a high incidence of pre-pregnancy complications such as fetal loss (12-21 weeks), preeclampsia, placental detachment, small for gestational age and fetal birth, but without significant differences between the two groups.
Types of interventions	Group A: Low molecular weight heparin prescribed (enoxaparin sodium 0.2 ml, 20 mg once daily subcutaneously, Clexane^®^, Aventis Pharma, Egypt) from the time of ultrasonographic confirmation of fetal viability up to 34-week pregnancy and folic acid tablets 0.5 mg daily up to 13 weeks gestation. Platelet counts and activated partial thromboplastin time (usually 25-35 weeks) were performed 10 to 20 days from the start of treatment and every month thereafter until 34 weeks of gestation. Group B: Folic acid prescribed 0.5 mg tablets daily up to 13 weeks of gestation. Prenatal visits were made for all patients every 2 weeks until the first 20 weeks and then every 4 weeks until 36 weeks and then every week until delivery. During prenatal visits, obstetric ultrasound and laboratory investigation were performed for all patients.
Results measures	End of pregnancy and its results
Results	LMWH appears to be a safe and effective drug in significantly reducing the incidence of recurrent miscarriages of unknown etiology when administered in the first trimester and continued throughout pregnancy.

**Pasquier *et al*., 2015**

**Table:**

Authors	E. Pasquier, L.S.M, C. Bohec, C. Chauleur, F. Bretelle, G. Marhic, G. L. GaL, V. Debarge, F. Lecomte, C. D. Ziad, V. L. Saada, S, Douvier, M. Heisert, D. Mottier.
Journal	Journal Blood
Year of publication	2015
Country	France
Period	From April 4^th^, 2007 to October 31^st^, 2012
Study Designer	Prospective randomized study, double-blind
Randomization	A computer performed randomization, and eligible patients were randomly assigned to one of two groups.
Inclusion criteria	Pregnant women aged 18 to 45 years and history of unexplained recurrent abortion. The current pregnancy had to be confirmed by a clinician. Recurrent spontaneous abortion was defined as ≥2 consecutive miscarriages before 15 weeks of gestation, conception with the same partner and no live births after consecutive miscarriages.
Exclusion criteria	Women with another indication for aspirin or anticoagulant therapy (e.g. increased risk of venous thromboembolism during pregnancy, chronic antithrombotic therapy for cardiovascular disease), contraindication to enoxaparin injections with 40 mg according to French labeling (e.g. anemia <10g/dL, platelet count <150 x 10^12^/L, creatinine clearance <30 mL / min), were either unwilling or unable to consent.
Participant demographic data	Women with an average age of 32 years (range 18-44). 72% of women had ≥ 3 previous miscarriages. The mean gestational age at randomization (i.e., the time the injections started) was 39 days of amenorrhea.
Types of interventions	Intervention group: prescribed enoxaparin 40 mg per day. Placebo group: prescribed saline solution. Enoxaparin and placebo were purchased from Sanofi-Aventis (ROVI branch for Placebo-Enoxaparin syringes, Madrid, Spain) and were packaged and labeled by the pharmacy clinical trial unit at Brest University Hospital. Enoxaparin and placebo were contained in identical syringes and packaged in identical sachets. Treatment was administered subcutaneously once daily, started from the inclusion visit (or within 24 hours) and continued by self-injection until 35 weeks of gestation. Women also received standard care and pregnancy monitoring with fetal ultrasound during the pregnancy. In addition, all were encouraged to take a folic acid supplement.
Results measures	The primary result measure was the rate of live and viable births. Secondary results were: spontaneous abortion rates, intrauterine fetal death after 20 weeks of gestation, preeclampsia, birth of a small neonate to pregnancy, placental detachment, premature birth, maternal thrombocytopenia rates (defined as platelet count <0.x basal platelet count or platelet count <100 000/mm^3)^, bleeding episodes and skin reactions.
Results	Enoxaparin administered at a daily dose of 40 mg did not improve the chance of live birth in non-thrombophilic women with a history of unexplained recurrent miscarriage. Enoxaparin use at a daily dose of 40 mg was safe during early pregnancy.

**Shaaban *et al*., 2017**

**Table:**

Authors	O. M. Shaaban, A. M. Abbas, K. M. Zahran, M. M. Fathalla, M. A. Anan and S. A. Salman
Journal	Thrombosis/Clinical and applied Hemostasis
Year of publication	2016
Country	Egypt
Period	From January 1^st^, 2011 to December 2014.
Study Designer	Prospective randomized study
Randomization	A computer performed randomization and eligible patients were randomly assigned to one of two groups.
Inclusion criteria	Pregnant women between 20 and 35 years with regular marital life with the same partner, regular menstrual cycle before current pregnancy, cutaneous conception and history of habitual abortion defined as 3 or more consecutive miscarriages before 20 weeks of gestation.
Exclusion criteria	Women with polycystic ovary syndrome, any endocrine abnormalities such as diabetes mellitus, thyroid disorders, history of abnormal uterine cavity proven by hysterosonography or hysteroscopy before pregnancy. Women with positive inbreeding. Women who refused to participate.
Participant demographic data	Women with an average age between 23 and 30 years and average of 3 previous abortions.
Types of interventions	Group 1: prescribed 500 mg (tablets) of folic acid (Mepaco-Medifood, Egypt) daily together with 0.4mg/kg sodium tinzaparin (Innohep 4500 IU; LEO Pharma A/S, Denmark) daily subcutaneous injections. Group 2 (control group: folic acid prescribed at the same dose only without LMWH.
Results measures	Primary Result: viable pregnancy continuation beyond 20 weeks of gestation. Secondary Result: Results were home-baby rate, spontaneous abortion rate, intrauterine growth restriction, preeclampsia, maternal hemorrhage, heparin-induced thrombocytopenia, injection site pain and bruising, teratogenicity.
Results	The use of LMWH (tinzaparin) decreased the rate of early and late miscarriage and increased the rate of live births, and LMWH treatment may increase the percentage of babies at home.

**Urman *et al*., 2009**

**Table:**

Authors	B. Urman, B. Ata, K. Yakin, C. Alatas, S. Aksoy, R. Mercan and B. Balaban
Journal	Human Reproduction
Year of publication	2009
Country	Turkey
Period	From January 2006 to May 2008.
Study Designer	Prospective randomized study
Randomization	Computer generated randomization prepared by one of the researchers. The study subjects were randomized in blocks of 10; that is, of every 10 randomized subjects, five were allocated to the LMWH arm and five were allocated to the control arm at random.
Inclusion criteria	-History of at least two transfer cycles of previously failed fresh embryos, as demonstrated with negative levels of human chorionic gonadotropin (hCG) in serum. -All previously failed cycles at the American Hospital in Istanbul. -Female age ≤38 years. -Fresh ejaculation sperm to be used for ICSI. No hormonal, clotting or immunological disorders have been detected. - Normal uterine cavity, assessed by hysteroscopy or serum-infused ultrasound. - Normal female and male peripheral karyotype
Exclusion criteria	Women requiring anticoagulant therapy for other medical reasons, obvious causes of implantation failure (transvaginal ultrasound visible hydrosalpinx, fibroids that distort the uterine cavity, absence of grade I, grade II embryos available for transfer or clinical or laboratory findings of congenital thrombophilia, or acquired.
Participant demographic data	Women aged between 29 and 41 years and with ≥2 failed assisted reproduction treatment cycles.
Types of interventions	Study group: LMWH (Enoxaparin Sodium, Clexane, Aventis Pharma) was administered at a dose of 1 mg/kg/day from the day after oocyte recovery. Control group: it received no medication other than progesterone gel.
Results measures	Primary result: continuous pregnancy rate. Secondary results: multiple pregnancy, implantation, continuous pregnancy at> 20 weeks, live births.
Results	The results of this pilot study suggest a potential beneficial effect of LMWH on the clinical outcome of assisted reproduction therapy (ART) in women with recurrent implantation failure (RIF). This finding, however, has yet to be corroborated by larger trials.

**Schleussner *et al*., 2015**

**Table:**

Authors	E. Schleussner, G. Kamin, G. Seliger, N. Rogenhofer, S. Ebner, B. Toth, M. Schenk, M. Henes,M. K. Bohlmann, T. Fischer, O. Brosteanu, R. Bauersach and D. Petroff
Journal	Annals of internal medicine
Year of publication	2015
Country	Germany and Austria
Period	From December 2006 to August 2012.
Study Designer	Multicenter randomized controlled trial using minimization.
Randomization	Randomization was stratified by gestation week using the minimization method described by Pocock and Simon.
Inclusion criteria	Women with at least 2 consecutive miscarriages (<12weeks gestation) or 1 late miscarriage (≥12 weeks gestational age) and had a viable pregnancy during 5 to 8 weeks of pregnancy detected by ultrasound.
Exclusion criteria	Women who had previous abortions due to chromosomal alterations, uterine structural abnormalities; women with diabetes mellitus; nicotine use, drugs, alcohol use, HIV infection; women judged by researchers as risk of poor adherence; women in clinical need of heparin therapy or any contraindication to LMWH; women with Leiden factor V homozygous mutations, prothrombin homozygous mutations, or antiphospholid antibody syndrome (lupus anticoagulant, anti-beta2-glycoprotein 1, or anticardiolipins).
Participant demographic data	Women with an average age of 32.3 years. 24% of women have had at least 1 late abortion (>12 weeks gestation) and 44% have had 3 or more miscarriages.
Types of interventions	Women from both groups received folic acid containing multivitamins (Femibion 800 Metabolism [Merck]) from allocation up to 24-week gestation. The women returned for study visits at 9, 12, 16, 20 and 24 weeks gestation. Intervention group: Each visit received 30 syringes each containing 5000 IU dalteparin sodium to be self-administered daily as a subcutaneous injection (Fragmin P Forte [Pfizer]) up to 24 weeks of gestation. Control group: received no placebo injections.
Results measures	Primary result: ongoing pregnancy rate up to 24 weeks gestation. Secondary results: live birth rate, preeclampsia, placental premature detachment, preterm delivery within 37 weeks of gestation, or intrauterine growth restriction below the 5thpercentile.
Results	Study data show that LMWH prescribed for women with recurrent unexplained pregnancy loss and a viable pregnancy does not increase live birth rates. In addition, the data show that any effect that LMWH can have is minimal. Given the daily injection weight, we do not recommend its use in such women with the purpose of reducing spontaneous abortion rates.

**Noci *et al*., 2011**

**Table:**

Authors	I. Noci , M. N. Milanini, M. Ruggiero, F. Papini, B. Fuzzi, P. G. Artini
Journal	Online Reproductive Biochemistry
Year of publication	2011
Country	Italy
Period	From May, 2008 to December, 2008
Study Designer	Prospective randomized study
Randomization	Not described
Inclusion criteria	Women aged <40 years without congenital or acquired thrombophilic state, absence of endocrine, hematologic abnormalities, chronic diseases, uterine pathology interfering with embryonic implantation.
Exclusion criteria	Women with endocrine, hematologic abnormalities, chronic diseases, uterine pathology, congenital or acquired thrombophilia.
Participant demographic data	Women with an average age of 31.1 to 38.3 years.
Types of interventions	Treatment group: In the luteal phase they received support with vaginal progesterone (Prometrium, 200mg twice daily) and a prophylactic dose of dalteparin sodium (Fragmin, 2500 IU s.c. daily; Pfizer Italy, Latina, Italy) in the afternoon of the day of recovery from oocytes until the day of the pregnancy test. Control group: luteal phase support was initiated with vaginal progesterone (Prometrium 200 mg twice daily; Rottapharm, Milan, Italy) from the following day oocyte recovery to the day of pregnancy testing (12 days after embryo transfer).
Results measures	Primary result: live birth rate. Secondary results: implantation rate and clinical pregnancy rate.
Results	Prophylactic LMWH administration to non-thrombophilic non-women who have undergone their first IVF cycle could increase the live birth rate, implantation rate, and clinical pregnancy rate. However, these changes are not significant and require a larger multicenter study.

**Berker *et al*., 2011**

**Table:**

Authors	B. Berker, S. Taskın, K. Kahraman, E. A. Taskın, C. Atabekoglu, M. Sonmezer
Journal	Fertility and Sterility
Year of publication	2011
Country	Turkey
Period	From June 2007 to October 2009.
Study Designer	Prospective, quasi-randomized controlled trial.
Randomization	Quasi-randomization
Inclusion criteria	Women with recurrent implantation insufficiency (RIF) but no coagulation disorders and who had previously had at least two consecutive failed cycles of intracytoplasmic sperm injection and embryo transfer (ICSI-ET), which we defined as RIF.
Exclusion criteria	Women with coagulation disorders, conditions requiring anticoagulant treatment, hydrosalpinx, lesions that distort the uterine cavity such as polyps or fibroids, lack of a grade I or II embryo for transfer, or without available sperm or oocyte.
Participant demographic data	Women with an average age between 26.4 and 36.2 years and with an average of 1.7 to 3.7 previously failed cycles.
Types of interventions	Study group: LMWH (sodium enoxaparin, Clexane, 4000 anti-Xa IU (40mg)/0.4 mL; Aventis Intercontinental, Paris, France) administered at a standard dose of 40 mg/0.4 mL per day from the day of the oocyte recovery. Patients self-administered LMWH subcutaneously and continued until 12 weeks of pregnancy if the pregnancy test 12 to 14 days after ET was positive.
Results measures	Primary result: live birth rate. Secondary results: implantation rate and clinical pregnancy rate.
Results	The results showed no beneficial effect of LMWH on pregnancy outcomes in patients with two or more implantation failures and no coagulation disorders. Pregnancy results were better in the LMWH subgroup of patients with three and more implantation failures, but were not statistically significant. The use of LMWH should be limited to the research objectives until its beneficial effects have been proven by studies.
